# A novel ammonia-oxidizing archaeon from wastewater treatment plant: Its enrichment, physiological and genomic characteristics

**DOI:** 10.1038/srep23747

**Published:** 2016-03-31

**Authors:** Yuyang Li, Kun Ding, Xianghua Wen, Bing Zhang, Bo Shen, Yunfeng Yang

**Affiliations:** 1 Environmental Simulation and Pollution Control State Key Joint Laboratory, School of Environment, Tsinghua University, 100084, Beijing, P.R. China

## Abstract

Ammonia-oxidizing archaea (AOA) are recently found to participate in the ammonia removal processes in wastewater treatment plants (WWTPs), similar to their bacterial counterparts. However, due to lack of cultivated AOA strains from WWTPs, their functions and contributions in these systems remain unclear. Here we report a novel AOA strain SAT1 enriched from activated sludge, with its physiological and genomic characteristics investigated. The maximal 16S rRNA gene similarity between SAT1 and other reported AOA strain is 96% (with *“Ca.* Nitrosotenuis chungbukensis”), and it is affiliated with Wastewater Cluster B (WWC-B) based on *amoA* gene phylogeny, a cluster within group I.1a and specific for activated sludge. Our strain is autotrophic, mesophilic (25 °C–33 °C) and neutrophilic (pH 5.0–7.0). Its genome size is 1.62 Mb, with a large fragment inversion (accounted for 68% genomic size) inside. The strain could not utilize urea due to truncation of the urea transporter gene. The lack of the pathways to synthesize usual compatible solutes makes it intolerant to high salinity (>0.03%), but could adapt to low salinity (0.005%) environments. This adaptation, together with possibly enhanced cell-biofilm attachment ability, makes it suitable for WWTPs environment. We propose the name “*Candidatus* Nitrosotenuis cloacae” for the strain SAT1.

Nitrification is a significant biological process for nitrogen removal in wastewater treatment plants (WWTPs). Ammonia oxidation, the first and rate-limiting step of nitrification, is critical for wastewater treatment^1^. For a long time, it has been believed that this step is solely mediated by ammonia-oxidizing bacteria (AOB), which are affiliated with *Betaproteobacteria* and *Gammaproteobacteria*^2^. In the last decade, the discovery and cultivation of ammonia-oxidizing archaea (AOA) in various environments has extended the boundary of ammonia-oxidizers to the domain *Archaea*^3^,^4^. The most important characteristic of AOA is their possession of archaeal *amoA* gene, which codes for the α-subunit of ammonia monooxygenase, the key enzyme responsible for ammonia oxidation. Using *amoA* as gene marker, recent investigations revealed that AOA occurred with great abundances in sites such as marine environment and acidic soils^5^,^6^.

In WWTPs, Park *et al*. was the first to report detection of AOA in five plants with low dissolved oxygen (DO) level and long retention time^7^, which suggested potential application of AOA to oxidize ammonia with less DO and consequently low energy consumption. Since then, the presence of AOA has been confirmed in a number of WWTPs worldwide, and the abundances of AOA even outnumbered that of AOB in some of those plants^8^,^9^,^10^. However, AOA was not widespread in WWTPs as AOB, as Mussmann’s study showed that AOA was only detected in 4 out of total 52 plants^9^. A list of operational and environmental parameters had been proposed as influential factors for occurrence and distribution of AOA in WWTPs, including ammonia, dissolved oxygen, salinity and retention time^7^,^8^,^11^,^12^, but their conclusions were often conflict with each other and there has not been a consent point of view. Current studies on AOA in WWTPs have mainly focused on AOA occurrence, abundance and diversity by using culture-independent methods such as quantitative PCR and high-throughput sequencing, combined with statistics tools like correlation analysis, which might lead to large bias due to the limitation of detection and complexity of WWTPs environment^13^,^14^. To know AOA precisely, which is the prerequisite of its application, cultivation of AOA from WWTPs is necessary. By focusing on a single strain, one could investigate its physiological characteristics through more accurate experiments.

To date, a number of AOA has been enriched or isolated from various environments, including marine environment^4^,^15^, hot spring^16^,^17^, neutral soil^18^,^19^, acidic soil^20^ and freshwater^21^, with their whole genome sequence nearly all obtained^22^. However, no AOA enriched from WWTPs has been reported. In this study, we successfully enriched a novel ammonia-oxidizing archaeon strain SAT1 from a full-scale municipal WWTP, with its physiological characteristics like cell morphology, growth rate, mode of nutrition, and influence factors investigated. Moreover, we obtained its completed genome sequence, and predicted its potential functions based on genome annotations. This strain is affiliated with the previously proposed wastewater cluster B^7^, a cluster specific for activated sludge reactors and is detected frequently in different WWTPs. The strain SAT1, as a representative AOA from wastewater, the research findings on it will extend our understanding on AOA occurrence and functions in WWTPs and may lay foundation for their future application.

## Results

### Establishment of highly purified AOA enrichments

By using activated sludge from a full-scale WWTP as initial inoculums, AOA enrichment culture was established. Antibiotics including 50 mg/L ampicillin and 25 mg/L streptomycin were used. A long time (about 10 weeks) was needed for complete consumption of about 0.3 mM ammonia in initial cultivation, but it was shortened to about 5 weeks after the second transfers (Supplementary Fig. S1). The successful PCR amplification of archaeal 16S rRNA and *amoA* gene indicated the presence of AOA. After 5–6 transfers, antibiotics were removed from the medium and filter-transferring was used, which further shortened the time of each cycle to 3–4 weeks. After near one year’s enrichment, the culture was used for further analysis.

The absence of AOB in the cultivation system was suggested by negative amplification of bacterial *amoA* gene using primers (*amoA*1F/*amoA*2R, see Supplementary Table S1). Thirty five nearly identical (with sequencing and amplification errors less than 3 nucleotides) archaeal 16S rRNA sequences were identified from the constructed clone library, indicating that only one archaeon was obtained, here referred to as strain SAT1. The uniformity of archaeal 16S rRNA was also checked by Denaturing Gradient Gel Electrophoresis (DGGE) analysis, resulting in only one single band obtained (Supplementary Fig. S2).

The purity of AOA was analyzed by the quantification of archaeal and bacterial 16S rRNA gene within the SAT1 enrichments. Quantitative PCR showed that the archaea 16S rRNA gene consist of 91% of total prokaryotes ((6.68 ± 1.92) × 10^8^ for archaea and (6.25 ± 0.77) × 10^7^ for bacteria), and metagenomic data showed that the ratio was 85% (459 archaeal and 83 bacterial 16S rRNA sequences based on shotgun sequencing and RDP classification), indicating that the strain SAT1 was highly enriched. Attempts to isolate pure strain failed, and the contaminated bacteria were analyzed by 16S rRNA clone library. The dominated bacteria were associated with the genera *Ralstonia* (90%), *Afipia* (3%), *Ohtaekwangia* (3%) and *Tardiphaga* (3%). No AOB or nitrite-oxidizing bacteria (NOB) 16S rRNA sequences were obtained.

**Figure 1:**
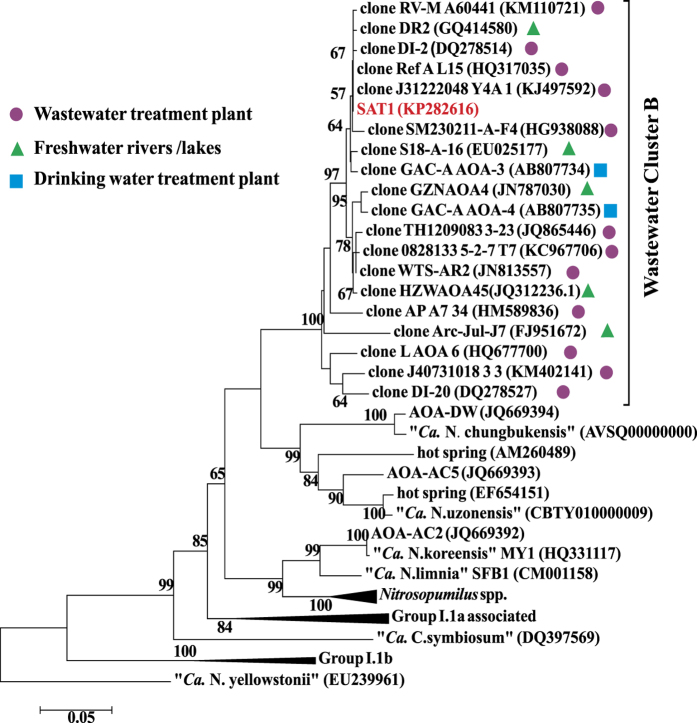
Phylogenetic tree showing the relationships of *amoA* gene sequence of strain SAT1 to reference sequences from the GenBank database. The tree was constructed with the neighbor-joining method. Bootstrap values shown at nodes where the value was greater than 50, are based on 1000 trials. For sequences inside wastewater cluster B, those from wastewater treatment plant were marked with circles (●), those from freshwater rivers/lakes were marked triangles (▲), and those from drinking water treatment plant were marked block (■).

Based on 16S rRNA sequence, the strain SAT1 is affiliated with Group I.1a of the phylum *Thaumarchaeota* (Supplementary Fig. S3). The maximum similarity between SAT1 and other reported AOA strain is 96% (with *“Ca.* Nitrosotenuis chungbukensis”), indicating that SAT1 is a novel strain^23^. The *amoA* gene phylogeny of the strain SAT1 is congruent with that of 16S rRNA gene, and it is also affiliated with Wastewater Cluster B (WWC-B), a cluster specific for activated sludge reactors^7^ (Fig. 1).

**Figure 2:**
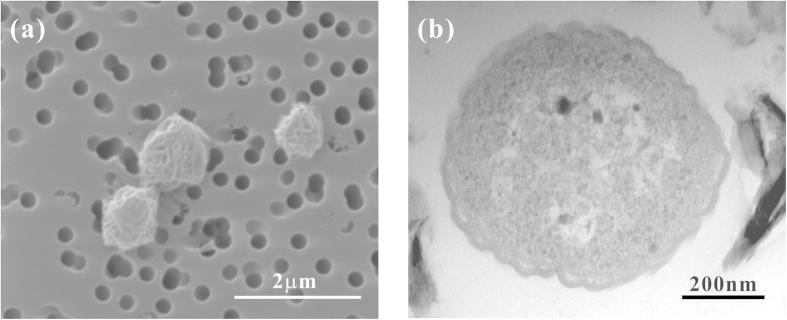
Photomicrographs of the SAT1 enrichment culture using SEM (**a**) and TEM (**b**).

Unexpectedly, The SAT1 cells were spherically shaped based on SEM and TEM analyses, with diameter of 1.1 ± 0.1 μm (Fig. 2). The cell shape of SAT1 is similar to that of Group I.1b AOA^18^,^24^, but different from other Group I.1a strains, which were all rod shaped^4^,^17^,^25^,^26^.

### The growth and autotrophy of the strain SAT1

**Figure 3:**
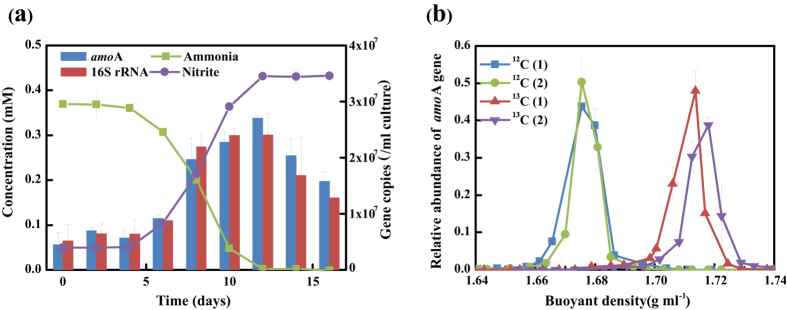
Autotrophic growth and ammonia oxidation by strain SAT1. (**a**) Cell growth is represented by archaeal *amoA* and 16S rRNA gene abundance. Error bars represent the standard deviations from triplicate experiments. (**b**) Distribution of the relative abundance of archaeal *amoA* gene in CsCl gradient for ^13^C-NaHCO_3_, or ^12^C-NaHCO_3_ treatment. The number in parentheses (1 or 2) means the cycles of incubation. Vertical and horizontal error bars represent standard deviations of the relative abundance and buoyant density of fractions from duplicate cultivation samples respectively.

The growth curve of strain SAT1 were demonstrated by its cell abundance together with the decrease of initial ammonia concentration coupled to exponential increases of nitrite concentration (Fig. 3a). The cell abundances were represented by archaeal 16S rRNA and *amoA* gene copies detected by quantitative PCR. The maximum growth rate, estimated from 16S rRNA gene abundance, was 0.25 d^−1^ (with doubling time of 2.9 d), which was comparable to that of *Nitrososphaera* sp. JG1^24^, but lower than most of the other AOA strains. The cell ammonia oxidation activity was estimated as 3.8 fmol cell^−1^ d^−1^, which was high than that of *Nitrososphaera* sp. JG1 (1.4 fmol cell^−1^ d^−1^) and *“Ca.* Nitrosoarchaeum koreensis” (2.5 fmol cell^−1^ d^−1^), but lower than that of *Nitrosopumilus maritimus* (12.8 fmol cell^−1^ d^−1^).

The carbon fixation ability of strain SAT1 was determined using stable isotope probing (SIP). Duplicated incubations were performed for 2 cycles of cultivation (approx. 4 weeks per cycle) in the presence of 2.5 mM ^12^C-NaHCO_3_ or ^13^C-NaHCO_3_. Ammonia oxidation was indicated by decreases in ammonia concentration and increases in nitrite concentration and *amoA* gene copies as usual cultivation without NaHCO_3_. After completely consumption of ammonia, the media were used for genomic DNA extraction and gradient centrifugation. The relative proportion of archaeal *amoA* gene copies in CsCl gradients was determined by quantitative PCR of three replicates. The archaeal *amoA* genes reached the maximum value around a buoyant density of 1.67 g ml^−1^ under ^12^C-NaHCO_3_ treatment for both of the 2 cycles’ incubation. For ^13^C-NaHCO_3_ treatment group, the maximum archaeal *amoA* genes value shifted to around a buoyant density of 1.71 g ml^−1^ after the first cycle of incubation, and further shifted even heavier after the second cycle (Fig. 3b). Although the buoyant densities as a whole were lower than the standard ones, a difference of 0.04 g ml^−1^ fitted well with those between unlabeled and fully labeled ^13^C-DNA^27^. Thus, the ability of incorporating ^13^C-NaHCO_3_ into DNA indicated that the strain SAT1 could grow autotrophically.

### The influences of environmental factors on strain SAT1

**Figure 4:**
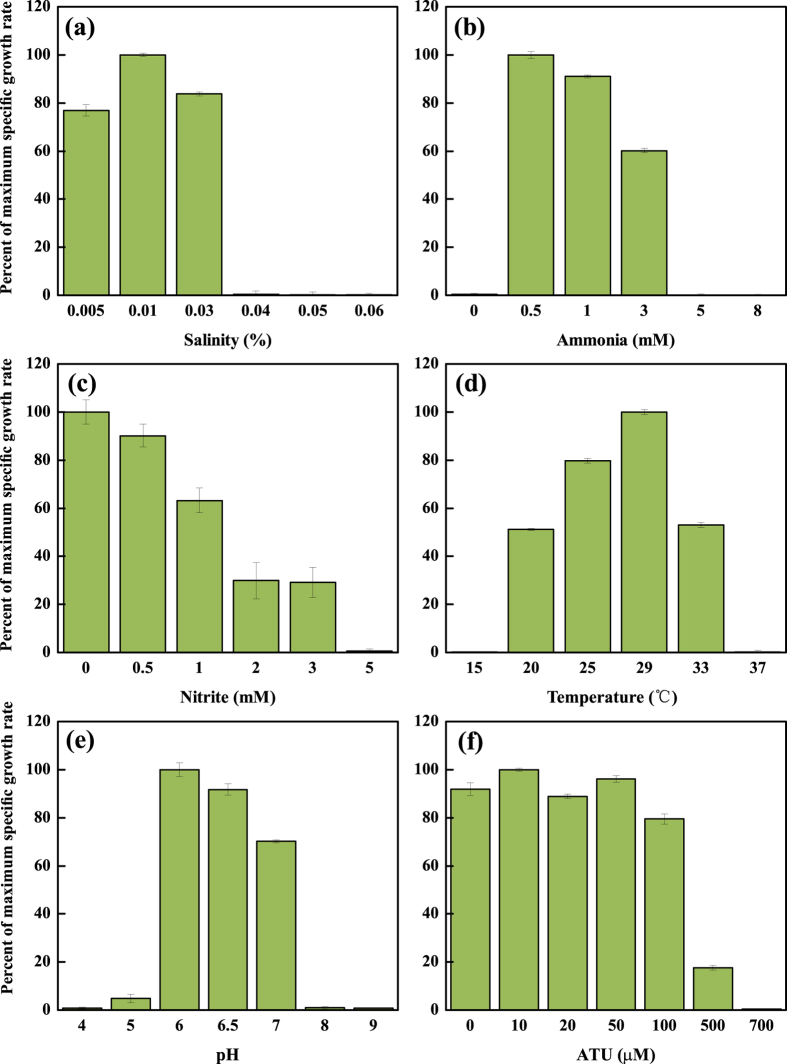
Influence of (**a**) salinity, (**b**) ammonia, (**c**) nitrite, (**d**) temperature, (**e**) pH and (**f**) Allylthiourea on the growth activity of strain SAT1. Values represent percentage of specific growth rates of cultures grown under different environmental values relative to those at optimal ones. Error bars represent the standard deviations of triplicate cultures.

For each environmental factor, the influences were determined by comparing the specific growth rates of AOA under gradient conditions. As for salinity, gradient concentrations of NaCl ranging from 0.005%–0.1% were used. It was shown that the strain SAT1 could adapt only to salinity no higher than 0.03% (~647 μS cm^−1^ in electrical conductivity) (Fig. 4a). It had a much lower salinity tolerance than any other reported AOA strain such as *“Ca.* Nitrosoarchaeum koreensis” (0.4%)^25^ and *Nitrosopumilus maritimus* (>3.5%)^15^. However, strain SAT1 was tolerant to salinity as low as 0.005% (~90 μS cm^−1^ in electrical conductivity), which was even lower than that of common wastewater. This value was comparable to that of *“Ca.* Nitrosotenuis uzonensis” (0.005%)^17^, and far below the lower limit of *“Ca.* Nitrosoarchaeum koreensis” (0.1%)^25^. These results indicated that the strain SAT1 was nonhalophilic, and it could easily adapt low salinity environment. The optimal salinity was 0.01%.

The growth of stain SAT1 was inhibited by ammonia or nitrite higher than 3 mM (Fig. 4b,c). This tolerant limits were comparable with *Nitrosopumilus maritimus*^4^ and *“Ca.* Nitrososphaera gargensis”^28^, but lower than that of *Nitrosotalea devanaterra* (up to 50 mM)^20^. The strain SAT1 was adapted to temperature from 25 °C to 33 °C, and the optimum was 29 °C (Fig. 4d). The pH range was 5.0 to 7.0, with the optimum pH at 6.0 (Fig. 4e). These properties indicated that the strain SAT1 was mesophilic and neutrophilic.

The inhibition of allylthiourea (ATU) to the SAT1 was tested. No inhibition was observed when ATU concentration was lower than 100 μM, partial inhibition occurred at 500 μM, and complete inhibition at 700 μM (Fig. 4f). This inhibition concentration for the SAT1 was high, only comparable to that of *“Ca.* Nitrosoarchaeum koreensis” (>500 μM)^25^, and much higher than other AOA strains (100 μM for *Nitrososphaera* sp. JG1^24^, and 50 μM for *“Ca.* Nitrosotenuis chungbukensis”^26^) and AOB (<10 μM^29^). Since ATU could chelate Cu^2+^, the higher inhibition concentration demonstrated that the strain SAT1 had a higher affinity for Cu^2+^, which might result from the high content of copper-containing protein in its genome (see below).

### The general features of SAT1 genome

High-throughput sequencing based on 500 bp and 5-kb libraries was performed. After sequence filtration, assembling and gap closing, a complete genome of the strain SAT1 was reconstructed (see Supplementary Fig. S4). The genome size was 1620156 bp, including 1717 protein coding sequences (CDS), 3 rRNAs, 41 tRNAs and an average GC content of 41.0%. Other general features of the SAT1 genome in comparison with those of other AOA and *Nitrosococcus oceani* (AOB) were listed in Supplementary Table S2. The 16S rRNA and *amoA* gene sequences in this genome were nearly identical (with sequencing and amplification errors less than 3 nucleotides) to that obtained from clone library before.

When aligning genomes using MUMmer 3.0, similar genome syntenies were observed between the SAT1 and other Group I.1a AOA except “*Ca.* Cenarchaeum symbiosum”, no large syntenic regions were observed between the SAT1 and Group I.1b genome (Supplementary Fig. S5). It was noted that there was a remarkable inversion of large DNA fragment in the genome of SAT1 when comparing with Group I.1a strains (Supplementary Fig. S5a), which was never found in other AOA genomes. The inverted fragment was from the site of around 0.3 Mb to 1.4 Mb, about 1.1 Mb in size, and accounted for 68% of the entire genome. It might be caused by recombination during the evolution of the strain SAT1. Similar large fragment chromosome inversions were also observed in some other bacterial strains like *Staphylococcus aureus*^30^ and *Bacillus anthracis* CDC 684^31^, which resulted in their reduced cell growth compared with closer strains without inversion. Thus, it was likely that the relatively low growth rate of the strain SAT1 was caused by the chromosomal inversion.

Comparing to other known AOA strains, the SAT1 had the largest genomic average nucleotide identity (ANI) of 75.49% with *“Ca.* Nitrosotenuis chungbukensis” and 75.11% with *“Ca.* Nitrosotenuis uzonensis” (Supplementary Table S4), which is far below the threshold for species demarcation (95–96%)^32^. Since ANI values below 75% are not to be trusted, the average amino acid identity (AAI) was calculated based on protein sequence, resulting in lower identities: 68.88% with *“Ca.* Nitrosotenuis chungbukensis” and 73.76% with *“Ca.* Nitrosotenuis uzonensis” (Supplementary Table S4). These evidences together with the genomic phylogeny (Supplementary Fig. S6), suggested that strain SAT1 was a novel AOA species within the genus of “*Ca.* Nitrosotenuis”.

### Nitrogen metabolism characteristics of the strain SAT1

Putative genes encoding ammonia monooxygenase were found in the genome of the SAT1 with its subunits arranged in the same order (*amoB-amoC-amoX-amoA*, Supplementary Fig. S7; SU86_008310-SU86_008325 in Supplementary Data S1) as other Group I.1a AOA^22^,^26^,^33^. Additionally, two ammonia transporters genes (*amtB,* SU86_004840 and SU86_005670) were identified.

As all other known AOA, no homologs for bacterial hydroxylamine oxidoreductase (HAO) gene were identified in the genome of the strain SAT1. However, as proposed by Walker *et al*., numerous multicopper oxidases (MCOs) and small blue copper-containing proteins might take the roles of the missing HAO and cytochrome c protein, respectively^22^, thus suggest an alternative ammonia oxidation and electron transfer pathway via hydroxylamine^34^. Two MCOs were identified in the genome of the SAT1, and one of which (SU86_009190) was NO-forming nitrite reductase protein (NirK). NirK was also involved in the reduction of NO_2_^-^ to NO, and probably play an important role in N_2_O production. But the downstream genes, encoding nitric oxide reductase subunits (NorD/NorQ), which were present in the genome of *“Ca.* Nitrososphaera evergladensis”, were absent in that of SAT1. Thus production of N_2_O by the strain SAT1 remains elusive. Besides, seven small blue copper-containing proteins were identified (Supplementary Data S1). One of which (SU86_000100) contained three cupredoxin domains, which might be a candidate for quinone reductase (QRED) as in *Nitrosopumilus maritimus*.

**Figure 5:**
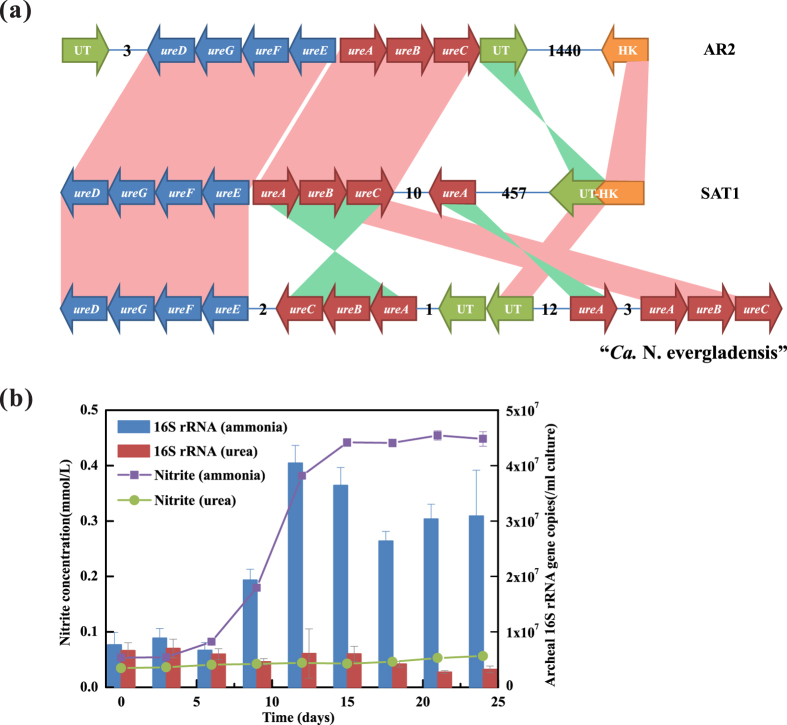
(**a**) Organization of urease utilizing genes of SAT1 genome and (**b**) the experimental test of its urea utilizing ability. The organization of genes was in comparison with *“Ca.* Nitrosopumilus sediminis” AR2 (above) and *“Ca.* Nitrososphaera evergladensis” (below). Urease core protein genes were shown in red, urease accessory protein genes were shown in blue, urea transporter (UT) genes were shown in green, and histidine kinase (HK) genes were shown in orange. For comparison, forward matches colored in red and reverse matches colored in green. The numbers between two genes indicated the number of interval ORFs. For the test of the urea utilizing ability of SAT1, the nitrite production and archaeal 16S rRNA abundance during one cycle of incubation, using 0.5 mM NH_4_^+^ (purple line and blue column) and 0.25 mM Urea (light green line and red column) as substrate respectively.

Urea was an alternative nitrogen and energy source for some AOA strains such as *Nitrososphaera viennensis*^18^, *“Ca.* Nitrososphaera gargensis”^35^ and “*Ca.* Cenarchaeum symbiosum”^36^, with urease and urea transporter gene identified in their genome (Supplementary Table S3). For the SAT1 genome, genes coding for urease core proteins (*UreC, UreB, UreA,* locus tag in Supplementary Data S1) and urease accessory proteins (*UreE, UreF, UreG, UreD*) were identified (Fig. 5a). However, no completed urea transporter gene was found, except that a pseudogene (SU86_09525), 457 ORFs far from urease gene cluster, was identified as a combination of a C-terminal truncated urea transporter and an N-terminal truncated histidine kinase. This truncated urea transporter, which was caused by gene combination during evolution, might have already lost its functions. To confirm this, we use urea, instead of ammonia, as substrate in cultivation of AOA. After one cycle of normal incubation (about 25 days), no increase on the nitrite concentration or cell abundance was observed comparing with those using ammonia as substrate (Fig. 5b), and it remained the same when further extending the incubation time to two months. These results indicated that the strain SAT1 had lost the ability of urea utilization due to the truncation of urea transporter gene.

### Carbon metabolism characteristics of the strain SAT1

In agreement with all of other thaumarchaeal genomes^22^,^35^, the SAT1 genome contained all key genes for 3-hydroxypropionate/4-hydroxybutyrate (3-HP/4-HB) cycle (Supplementary Data S1), which was responsible for autotrophic carbon fixation. This cycle was a modified version of the autotrophic hydroxypropionate/hydroxybutyrate cycle of *Crenarchaeota*, and believed to be the most energy efficient pathway than any other aerobic autotrophic pathway^22^,^37^,^38^.

Reductive tricarboxylic acid (TCA) cycle was also used by some autotrophic bacteria for carbon fixation. A near complete reductive TCA cycle could be reconstructed based on related genes of the SAT1 genome (Supplementary Data S1). However, the conversion of citrate to oxaloacetate is uncertain. This step, the so called citrate cleavage, could be accomplished by three ways: catalyzed by citrate lyase (CL, EC 4.1.3.6), ATP citrate lyase (ACL, EC 4.1.3.8), or citryl-CoA synthetase (CCS) followed by citry-CoA lyase (CCL)^39^. No homologs for genes encoding enzymes for the latter two ways were identified in the SAT1 genome. For the first enzyme CL, it was composed of three subunits: α (*citF*), β (*citE*) and γ (*citD*), of which only *citE* (SU86_005245) was identified. Whether any unknown proteins in the genome of the SAT1 could substitute the function of *citF* and *citD*, and whether the strain SAT1 was capable of fixing CO_2_ via reductive TCA cycle need further investigation.

Two key genes coding for subunits of polyhydroxyalkanoate (PHA) synthase were identified in the genome of SAT1: *phaC* (SU86_001140) and *phaE* (SU86_001145), and shared 48% and 28% similarities respectively with that in *“Ca.* Nitrososphaera gargensis”^35^. This enzyme could synthesize poly-β-hydroxybutyrate (PHB), the most common type of PHA, using (R)-specific-3-Hydroxybutanoyl-CoA as monomer^40^. However, in contrast to its enantiomer, (R)-3-Hydroxybutanoyl-CoA was not involved in thaumarchaeal or crenarchaeal 3-HP/4-HB cycle^37^. It could only be converted from the inversion of (S)-3-Hydroxybutanoyl-CoA by an epimerase, or from crotonoyl-CoA, another 3-HP/4-HB cycle intermediate, by (3R)-3-Hydroxybutanoyl-CoA dehydratase as in *Rhodospirillum rubrum*^40^. In the genome of the SAT1, only homology of the latter enzyme was identified (*phaJ*, SU86_006950), and this enzyme was present only in *“Ca.* Nitrosoarchaeum limnia” BG20 and *“Ca.* Nitrosotenuis chungbukensis” MY2, not in other thaumarchaeal genomes. These results indicated that these strains might take a distinct PHA synthesis pathway using crotonoyl-CoA as starting materials, but it need to be experimentally confirmed.

### Other important genomic features of the SAT1

Genes coding for flagella, chemotaxis and pili were identified in the genome of the SAT1 (Supplementary Data S1). Similar to *“Ca.* Nitrosoarchaeum limnia” SFB1, five copies of flagellin subunit coding genes (*flaB*) were found in the SAT1 genome, while only 1–2 copies were observed in other AOA genome^19^,^35^. However, we did not observe defined flagella from the strain SAT1 using TEM. Since flagella were relatively large motility organelles for the cell, the formation of the flagella and the expression of their components were regulated by environmental conditions^41^. Lower expression of flagella in certain environments had been reported in some prokaryotes like *Vibrio spp.* and *Escherichia coli*^42^,^43^. Thus, it was possible that the expression level of flagella genes of strain SAT1 was low in our lab environment, which made its flagella hard to be observed. To prove this, further studies on the morphology of strain SAT1 in WWTPs environments are needed in the future.

Various genes related to heavy metal and antibiotics resistant were identified in the SAT1 genome (Supplementary Data S1), while no CRISPR region was found. Heavy metal included copper, arsenic, tellurium, cobalt, zinc, and cadmium could be pumped out of the cell by related transporters. Among them, the cobalt-zinc-cadmium efflux system protein (SU86_005285) was non-homologous to the same efflux protein occurred in *“Ca.* Nitrososphaera evergladensis”^19^, and absent from other AOA strains. Antibiotic resistance proteins included transporters and potential antibiotic degradation enzymes. Interestingly, a gene coding for cephalosporin hydroxylase was found, which was absent from other cultivated AOA strains, and was only found in the single-cell extracted AOA *“Ca.* Nitrosoarchaeum limnia” BG20 and a metagenomic shotgun sequencing project *thaumarchaeote* SCGC AAA799-P11 (JOSZ00000000, unpublished). Estimated from the phylogenetic tree (Supplementary Fig. S8), this hydroxylase might be horizontally transferred from *Cyanobacteria* or *Pseudomonas* spp. Since the strain SAT1 was the only cultivated AOA possessed this gene, potential cephalosporin degradation activity should be examined in the future.

## Discussions

The SAT1 was the first cultivated AOA strain from WWTPs, which was affiliated with Wastewater Cluster B (WWC-B) based on its *amoA* gene phylogeny. This cluster was firstly proposed by Park *et al*.^7^, and AOA of this cluster was detected in a list of WWTPs across the world (Supplementary Table S5), suggesting a significant role in nitrogen removal in those plants. By far, the strain SAT1 was the only AOA enrichment of WWC-B, thus the study on its physiology and genome can greatly improve our understanding on the occurrence and function of this cluster in WWTPs. Based on the results above, it was possible that there were some intrinsic features made strain SAT1 suitable for WWTPs environment.

The first significant physiological feature of strain SAT1 shown in this study was its intolerance to high salinity. This could be partially explained by the genomic data. Most prokaryotes coped with high salinity (osmosis) by synthesizing compatible solutes, which were usually some highly soluble organic molecules that could help balance the intra- and extra-cellular osmotic pressure. In archaea, a list of compatible solutes was used for high osmosis response, including ectoine/hydroxyectoine, mannosylglycerate, di*-myo*-inositol phosphate (DIP), etc.^44^,^45^. Ectoine/hydroxyectoine were synthesized from L-aspartate-β-semialdehyde, with four enzymes (EctB, EctA, EctC and EctD) participating in that process (Supplementary Fig. S9a)^46^. Genes coding for these four enzymes were all found in the genome of *Nitrosopumilus maritimus*^22^ (Supplementary Table S3), which facilitated its adaptation to salinity higher than 3.5%, but absent from the genome of the SAT1. Mannosylglycerate could be used for high osmosis and heat stress protection, and it is synthesized from 3-phosphoglycerate and GDP- mannose via a two-step pathway (Supplementary Fig. S9b)^47^. Two genes responsible for this pathway were identified in *“Ca.* Nitrososphaera gargensis” and *“Ca.* Nitrososphaera evergladensis”^19^,^35^, but not in the genome of the SAT1. DIP was the third compatible solute used by Group I.1b AOA, its complete synthesis required four enzymes (Supplementary Fig. S9c)^19^,^48^. However, only the first enzyme was identified in the genome of the SAT1, thus made this pathway infeasible. On the other hand, the strain SAT1 could live in low salinity environment (0.01%, Fig. 4), which might result from the containing of large-conductance mechanosensitive channel (MscL) gene (SU86_08200) in its genome. When cells were in hypo-osmosis environment, the MscL channel could pump hydrated solutes, e.g. potassium, out of the cell. This caused a rapid decrease in cytoplasmic hydrated solutes concentrations, lowered the osmotic driving force for water entry, and protected the cells against lysis^49^. This channel was present in the genomes of a nearby low-salinity adapted AOA *“Ca.* Nitrosotenuis uzonensis” and another low salinity strain *“Ca.* Nitrosoarchaeum limnia” SFB1^17^, but absent from the genome of *Nitrosopumilus* spp. strain, which lived mainly in high-salinity marine environment^22^,^33^,^50^. Therefore, due to lack of key genes for three usual compatible solutes synthetic pathways and the presence of MscL channel, the living conditions of strain SAT1 were restricted to low salinity environment. For WWTPs, they were usually low in salinity (defined as <1% according to Kargi *et al*.^51^), except those for contaminated groundwater and in filtration of sea water, which would be suitable for strain SAT1. Similar salinity tolerant values and mechanisms might be used by WWC-B cluster strains, since most of them also lived in low-salinity environments like freshwater rivers and lakes as shown in Fig. 1, and they were phylogenetically related to the strain SAT1.

Another important feature which helped strain SAT1 adapted to WWTPs environment might be their possession of flagella. Besides cell mobility, archaeal flagella and pili played a central role in surface adhesion and the formation of cell-cell connections, and thus could facilitate the formation of biofilms as observed in *Euryarchaeota* strains like *Pyrococcus furiosus* and *Methanocaldococcus villosus*^52^,^53^. Although not as dense as those two *Euryarchaeota* strains, the flagella of *Thaumarchaeota* might also contribute to biofilm formation, which was especially significant for their survival in WWTPs. The relative low growth rate of *Thaumarchaeota* suggested that they could easily be washed out from WWTPs if lived dispersedly. On the contrary, if attached to the biofilms, the cell loss would be slow. This was evident from the low diversities of AOA communities in WWTPs. Several surveys had demonstrated that Group I.1b (mainly wastewater cluster D) AOA were the most dominant in WWTPs, then was WWC-B within Group I.1a, but *Nitrosopumilus* spp. AOA were rarely found^7^,^10^,^13^. Taking genomic properties into consideration, the former two clusters all contained genes coding for flagella and pili^17^,^19^,^26^,^35^, while *Nitrosopumilus* spp. AOAs were lack of them^22^,^33^,^50^. It was possible that due to the lack of flagella and pili, *Nitrosopumilus* spp. AOA lived dispersedly, independent of biofilms, just as they did in marine environments, which rendered them difficult to retain in WWTPs with relatively higher flow rate. Moreover, WWC-B strains were usually found in plants with biofilm process or granule sludge process (Supplementary Table S5). That might come from the easier biofilm formation abilities of this cluster, which would extend their retention time.

In summary, the low salinity adaptation and flagella and pili might help strain SAT1 adapt to artificial WWTPs environment. Similar mechanisms might be used for the nearby WWC-B strains. Once entered the biological reactors along with the wastewater influent, those strains would be able to stay and grow there and through artificial selections finally became a significant AOA cluster in WWTPs. In addition, the AOA of WWC-B cluster was also found in drinking water treatment plants (Fig. 1). Thus, our study provided a representative strain of WWC-B for further revealing the characteristics and functions of AOA in those systems.

This study reports the physiological and genomic characterization of a novel ammonia-oxidizing archaea, strain SAT1, which is from thaumarchaeal group I.1a and enriched from wastewater treatment plant. We propose the following *Candidatus* status for this archaeon:

“Nitrosotenuis cloacae” sp. nov

### Etymology

clo.a’cae. L. n. cloaca sewer, the source of the organism.

### Locality

A municipal wastewater treatment plant from Tianjin, China.

### Diagnosis

A chemolithoautotrophic ammonia oxidizer affiliated with group I.1a of the phylum *Thaumarchaeota* within the domain *Archaea*. This archaeon is spherically shaped, mesophilic (25 °C–33 °C), neutrophilic (pH 5.0–7.0), intolerant to high salinity (>0.03%), but could adapt to low salinity (0.005%) environments. Its genome size is 1.62 Mb, with a large fragment inversion (accounted for 68% genomic size) inside, could not utilize urea due to truncation of the urea transporter gene.

## Methods

### Description of sampling sites

Activated sludge used for AOA enrichment was taken from a full-scale WWTP in Tianjin (38.94°N, 117.02°E), China. This plant was equipped with a modified full range simultaneous nitrification-denitrification (SND) process called biological double-efficiency process (BDP)^54^, with the treatment capacity of 1 × 10^4^ m^3^/d. The relevant parameters and descriptions are as follows: DO, 0.6 mg/L; influent NH_4_-N, 80–300 mg/L; effluent NH_4_-N, <1 mg/L; MLSS, 6000–8000 mg/L; SRT, >20 d; annual average electrical conductivity, 561 μS cm^−1^. Samples were kept inside of icebox during transportation to the lab, and used for incubation immediately.

### Cultivation of AOA

A total of 5 ml activated sludge were added as inoculum to 50 ml medium in 120 ml serum bottles, and then sealed with rubber stoppers and aluminum caps (CNW technologies, USA). The medium was composted of NH_4_Cl (0.5 mM) and KH_2_PO_4_ (0.1 mM), no NaHCO_3_ or other salt was necessary in usual incubation. After autoclaving, 50 μl trace element solution (1000×)^55^ was added. Antibiotics were used to select against the cocultured bacteria in the initial phase (the first 4 to 5 transfers) of the enrichment, including ampicillin (50 mg/L) and streptomycin (25 mg/L), and replaced by filter-transferring after stable AOA enrichment was obtained. The pH of the medium remained almost stable around 6.5 during cultivation. Enrichment cultures were incubated at 29 °C without shaking in the dark. Ammonia and nitrite levels were monitored weekly or biweekly using a salicylic acid assay and N-(1-naphthyl) ethylenediamine dihydrochloride assay respectively. When 80% of the ammonia was consumed, 10% of the culture was filtrated through filters with 0.45 μm pore size (Pall, USA) to exclude AOB and contaminated fungi^18^,^21^ and then further transferred to a 50 ml new medium.

### Cloning, sequencing, and phylogenetic analysis

Cells were harvested from the enrichment culture by passing through a 0.2 μm GTTP membrane (Millipore, USA), immediately frozen and stored at −80 °C until further analysis. DNA was extracted using a FastDNA SPIN Kit for soil (MP Biomedicals, USA) following the manufacturer’s protocol. The concentration of DNA was determined using Nanodrop spectrophotometer ND-1000 (ThermoFisher Scientific, USA). The bacteria and archaeal 16S rRNA and *amoA* genes were amplified by PCR using the primers listed in Supplementary Table S1 in the supplemental material. All the PCR conditions were as follows except bacterial *amoA* gene: 94 °C for 5 min; 35 cycles of 94 °C for 30 s, 55 °C for 30 s, and 72 °C for 60 s; and 72 °C for 10 min. For bacterial *amoA* gene, a condition of 94 °C for 2 min; 35 cycles of 94 °C for 40 s, 51 °C for 60 s, and 72 °C for 60 s; and 72 °C for 5 min was used. Cloning into pGEM-Teasy vector (Promega, USA) and sequencing with T7 and SP6 vector primers was performed using standard procedures. Phylogenetic analysis of archaeal 16S rRNA and *amoA* gene was performed by using MEGA version 5.2^56^, and the neighbor-joining tree was constructed using Maximum Composite Likelihood method. Bootstrap value of 1,000 replicates was set to estimate the reliability of phylogenetic reconstruction.

### Quantitative PCR

Archaeal and bacterial 16S rRNA and *amoA* genes copy numbers were quantified using an iCycler iQ5 thermocycler (Bio-Rad, USA), with the primer listed in Supplementary Table S1. Quantification was performed in 25 μl volume containing 12.5 μl SYBR Premix Ex Taq (Takara, Japan), 0.4 μM of each primer and 0.2 mg mL^−1^ BSA. The thermal cycling conditions for archaeal and bacterial 16S rRNA genes were as follows: 95 °C for 5 min; 40 cycles of 95 °C for 10 s, 51 °C for 30 s, and 72 °C for 30 s; and 72 °C for 30 s. The conditions for archaeal *amoA* gene were similar, except for using 55 °C as annealing temperature. Readings were taken between each cycle. Standard curves were prepared using pGEM-T Easy Vector ligated with reference genes (KP282616 for archaeal *amoA* gene, KP004891 for archaeal 16S rRNA gene, and KR815497 for bacterial 16S rRNA gene), as previously described by Ding *et al*.^57^. The efficiencies of all the assays ranged from 95.6% to 103.4% with the R^2^ at least 0.991. The specificity of quantitative PCR was checked by analyzing the melting curves.

### Stable isotope probing fractionation

For SIP incubation, AOA subcultures were additionally supplemented with 2.5 mM NaHCO_3_ (^12^C or ^13^C), and pH was adjust to 6.5 as normal cultivation using 1 M HCl. The NaH^13^CO_3_ (99 atoms%) was purchased from Sigma-Aldrich (USA) Co. After one cycle of incubation, 10% of the medium was used as inoculation for a new cycle, and the rest were all used for DNA extraction. Two cycles of incubation with two replicates were performed totally. Extracted DNA (~2 μg) was added into CsCl gradients with an initial density of 1.696 g ml^−1^. Density gradient centrifugation was performed in 4.9 ml OptiSeal polyallomer tubes (Beckman Coulter, USA) in a VTi 90 vertical rotor, subject to centrifugation at 56200 rpm for 24 h at 20 °C^58^. Centrifuged gradients were fractionated into 24–25 equal volumes (~200 μl) as described in detail by Zhang *et al*.^59^. Nucleic acids were precipitated by using PEG 6000, and then dissolved in 30 ml of TE buffer.

### Electron microscopy analyses

For scanning electron microscopy (SEM) analysis, AOA cells were harvested using 0.22 μm polycarbonate GTTP membrane (Millipore, USA), and then fixed in 2.5% (vol/vol) glutaraldehyde in 0.1 M sodium phosphate buffer (pH 7.2) for 24 h at 4 °C, and dehydrated using a graded ethanol series (70 to 100%) and finally 100% tertiary butanol. The samples were examined with a Quanta 200 electron microscope (FEI, the Netherlands). For transmission electron microscopy (TEM) analysis, cells were collected from 500 ml of culture by centrifugation at 6000 g for 10 min, and then negatively stained with 1% (wt/vol) phosphotungstic acid. Ultrathin sections were produced with an EM UC6 ultramicrotome (Leica, Germany), stained with uranyl acetate and lead citrate, and examined with an H-7650B electron microscope (Hitachi, Japan).

### Genome sequencing and analysis

DNA for genome sequencing was extracted using PowerSoil DNA Isolation Kit (MO BiO, USA) from 500 ml culture of strain SAT1. Two DNA libraries were constructed: a paired-end library with an insert size of 500 bp and a mate-pair library with an insert size of 5 kb. Both libraries were sequenced using an Illumina HiSeq2500 by PE125 strategy. Library construction and Sequencing was performed at the Beijing Novogene Bioinformatics Technology Co., Ltd. Illumina PCR adapter reads and low quality reads were filtered using in-house program. The archaeal and bacterial communities of Metagenomic DNA were analyzed by using MG-RAST online tools^60^. The filtered reads were assembled by SOAPdenovo^61^, which yielded 537 contigs in total. The contaminated bacterial contigs were removed through analyzing the correlation between sequencing depth and GC content (Supplementary Fig. S10), and aligning with known contaminated bacterial genomes (e.g. *Ralstonia pickettii* 12D). The remaining gaps were closed using PCR and Sanger sequencing, which finally resulted in one completed scaffold of 1.6 Mb. Gene prediction was performed on the SAT1 genome assembly by GeneMarkS^62^ with integrated model which combine the GeneMarkS generated (native) and Heuristic model parameters. Transfer RNA (tRNA) genes were predicted with tRNAscan-SE^63^, Ribosome RNA (rRNA) genes were predicted with rRNAmmer^64^. A whole genome Blast search (E-value less than 1e-5, minimal alignment length percentage larger than 40%) was performed against 3 databases. They are KEGG (Kyoto Encyclopedia of Genes and Genomes)^65^, COG (Clusters of Orthologous Groups)^66^, and NR (Non-Redundant Protein Database). Comparison of the SAT1 and other AOA genome was performed using MUMmer 3.0^67^. The average nucleotide identity (ANI) and average amino acid identity (AAI) was calculated online (http://enve-omics.ce.gatech.edu/ani/)^68^,^69^.

## Additional Information

**How to cite this article**: Li, Y. *et al*. A novel ammonia-oxidizing archaeon from wastewater treatment plant: Its enrichment, physiological and genomic characteristics. *Sci. Rep.* **6**, 23747; doi: 10.1038/srep23747 (2016).

## Supplementary Material

Supplementary Dataset 1

Supplementary Information
