# Supplementary material for: A novel ammonia-oxidizing archaeon from wastewater treatment plant: Its enrichment, physiological and genomic characteristics
Source: Sci Rep. 2016 Mar 31;6:23747. doi: 10.1038/srep23747 (PMC4814877; doi:10.1038/srep23747)
Supplement: Supplementary Information [file srep23747-s2.pdf]

# *Supporting information*

**A novel ammonia-oxidizing archaeon from wastewater treatment plant: Its enrichment, physiological and genomic characteristics**

Yuyang Li[1], Kun Ding[1], Xianghua Wen[1*], Bing Zhang[1], Bo Shen[1], Yunfeng Yang[1]

[1]*Environmental Simulation and Pollution Control State Key Joint Laboratory, School of Environment, Tsinghua University, 100084, Beijing, P.R. China*

*corresponding author

Xianghua Wen, School of Environment, Tsinghua University, Beijing 100084 CHINA

Tel: +86-10- 62772837;

Fax:+86-10-62771472

E-mail: xhwen@tsinghua.edu.cn

The supporting information includes:

Supplementary Table S1 to Table S5 (This file)

Supplementary Figure Legends (This file)

Supplementary Fig. S1 to Fig. S10 (This file)

Supplementary reference (This file)

Supplementary Data S1 (a separated MS EXCEL file)

## Tables:

### Table S1 Primers used in this study.

| Name | Sequence(5’-3’) | Target gene | Application | Reference |
|---|---|---|---|---|
| 20F | TTCCGGTTGATCCYGCCRG | Archaeal 16S rRNA | Detection and phylogeny | [1,2] |
| 1492R | GGYTACCTTGTTACGACTT | | | |
| 519F | CAGCMGCCGCGGTAA | Archaeal 16S rRNA | qPCR | [1,3] |
| 727R | GCTTTCRTCCCTCACCGT | | | |
| 23F | ATGGTCTGGCTWAGACG | Archaeal *amo*A | Detection, phylogeny and qPCR | [4,5] |
| 616R | GCCATCCATCTGTATGTCCA | | | |
| 27F | AGAGTTTGATCMTGGCTCAG | Bacterial 16S rRNA | Phylogeny | [1] |
| 1492R | GGYTACCTTGTTACGACTT | | | |
| bac518F | CCAGCAGCCGCGGTAAT | Bacterial 16S rRNA | qPCR | [6,7] |
| bac786R | CTACCAGGGTATCTAATC | | | |
| *amo*A1F | GGGGTTTCTACTGGTGGT | Bacterial *amo*A | Detection | [8] |
| *amo*A2R | CCCCTCKGSAAAGCCTTCTTC | | | |

**Table S2 Genomic features of strain SAT1 compared with other ammonia oxidizers.**

| **Genome** | **SAT1** | ***Nitrosopumilus maritimus*** | **"*Ca.* Nitrosotenuis uzonensis"** | **"*Ca.* Nitrosotenuis chungbukensis"** | **"*Ca.* Nitrososphaera evergladensis"** | ***Nitrosococcus oceani* ATCC 19707** |
|---|---|---|---|---|---|---|
| Affiliation | Group I.1a | Group I.1a | Group I.1a | Group I.1a | Group I.1b | AOB |
| Scaffold | 1 | 1 | 14 | 24 | 1 | 1 |
| Contigs | 1 | 1 | 14 | 24 | 1 | 1 |
| Size（Mb） | 1.62 | 1.65 | 1.65 | 1.76 | 2.95 | 3.48 |
| GC content | 41.0% | 34.2% | 42.2% | 41.7% | 50.1% | 50.30% |
| ORFs | 1855 | 1847 | 1999 | 2166 | 3555 | 3186 |
| ORF density (ORF/kb) | 1.15 | 1.12 | 1.21 | 1.23 | 1.21 | 1.28 |
| 5S rRNA | 1 | 1 | 1 | 1 | 1 | 1 |
| 16S-23S rRNA | 1 | 1 | 1 | 1 | 1 | 2 |
| tRNA | 41 | 44 | 41 | 43 | 39 | 45 |
| Plasmid | 0 | 0 | 0 | 0 | 0 | 1 |

**Table S3 Important physiological and genomic properties of stain SAT1 and other ammonia oxidizers.**

| **Strain** | **SAT1** | ***Nitrosopumilus maritimus*** | **"*Ca.* Nitrosotenuis uzonensis"** | **"*Ca.* Nitrosotenuis chungbukensis"** | **"*Ca.* Nitrososphaera evergladensis"** | ***Nitrosococcus oceani* ATCC 19707** |
|---|---|---|---|---|---|---|
| Affiliation | Group I.1a | Group I.1a | Group I.1a | Group I.1a | Group I.1b | AOB |
| Genome accession Number | CP011097.2 | CP000866.1 | CBTY000000000 | AVSQ00000000 | CP007174.1 | CP000127.1 |
| Salinity range | 0.005%-0.03% | >3.5% | 0.005%-0.1% | * | \\ | \\ |
| Cell shape | spherically | rod | rod | rod | spherically | Spherically/ ellipsoidal |
| urea utilization genes | Incomplete | × | × | × | Complete | Complete |
| Flagella related genes | √ | × | √ | √ | √ | √ |
| Ectoine synthesis gene | × | √ | × | × | √ | √ |
| Mannosylglycerate synthesis gene | × | × | × | × | √ | × |
| DIP synthesis gene | × | × | × | × | √ | √ |

* "\\" means not mentioned in reference paper, "√" means presence, and "×" means absence.

**Table S4 Genomic traits of strain SAT1 and other AOA strains.**

| Reference genome | ANI | AAI | 16S rRNA identity | *amoA gene* identity | AMO subunit α identity |
|---|---|---|---|---|---|
| *Nitrosotenuis chungbukensis* MY2 | 75.49% | 68.88% | 96% | 86% | 97% |
| *Nitrosotenuis uzonensis* N4 | 75.11% | 73.76% | 96% | 86% | 96% |
| *Nitrosoarchaeum limnia* | 75.46% | 63.24% | 92% | 83% | 98% |
| *Nitrosopumilus* sp. AR2 | 74.20% | 63.08% | 92% | 84% | 96% |
| *Nitrosopumilus maritimus* | 73.96% | 63.43% | 92% | 84% | 96% |
| *Cenarchaeum symbiosum* | — | 58.30% | 92% | 78% | 95% |
| *Nitrososphaera gargensis* | — | 50.38% | 85% | 73% | 83% |

**Table S5 Summary of the wastewater treatment plants in which wastewater cluster B AOA were found.**

| Study | Country/Region | Process | Representative sequence |
|---|---|---|---|
| [9] | USA | aerated-anoxic Orbal with SRT=20d,HRT=61h | DQ278514.1 |
| Merbt et al. unpublished | Spain | unknown | HG938088.1 |
| [10] | Taiwan | conventional activated sludge with SRT=10d, HRT=8h | HM589836.1 |
| [11] | UK | Trickling filter | HQ317035.2 |
| Wu et al, unpublished | Taiwan | bioreactor with HRT/SRT>10 d | HQ677700.1 |
| [12] | China | A/O MBR, BAF, MBR | JN813557.1 |
| [13] | China, Australia | A/A/O, Anammox | JQ865446.1 |
| Gao et al. unpublished | China | unknown | KC967706.1 |
| Gao et al. unpublished | China | unknown | KJ497592.1 |
| Ma et al. unpublished | China | Modified bio-contact oxidation process | KM110721.1 |
| Gao et al. unpublished | China | nitritation-ANAMMOX systems | KM402141.1 |

**Figures:**

Fig. S1 **The ammonia consumption of each cultivation cycle from the initial enrichment.** Each line represent one cultivation cycle. The ammonia concentrations were monitored every two or three weeks using a salicylic acid assay.

Fig. S2 **The changes of Denaturing Gradient Gel Electrophoresis (DGGE) profile for archaeal 16S rRNA gene during enrichment.** Lane 0 stand for enrichment sample from Day 0 (original sludge), lane 1 from Day 70, lane 2 from Day 104, lane 3 from Day 310, lane 4 from Day 331. The band with an arrow pointing to was sequenced and confirmed to be the 16S rRNA sequence of the strain SAT1.

Fig. S3 **Phylogenetic tree showing the relationships of 16S rRNA gene sequence of strain SAT1 to reference sequences from the GenBank database.** The tree was constructed with the neighbor-joining method using about 1400 nucleotide positions. Bootstrap values shown at nodes where the value was greater than 50, are based on 1000 trials.

Fig. S4 **Circular representation of the genome of strain SAT1**. The outermost two circles were forward (circle 1) and reverse (circle 2) gene components shown in arcs, including CDS (blue), tRNA (light red) and rRNA (light purple). Circle 3 showed the blast results in comparison with *N.maritimus*, using program tblastx, with an alignment cutoff >15, an identity cutoff >40, and an e-value $<10^{-5}$. Circle 4 showed the GC content and circle 5 showed the GC skew, values greater or smaller than the average percentage in the overall chromosome are shown in green and purple respectively. The circular plots were drawn by using CGView server (http://stothard.afns.ualberta.ca/cgview_server/, Grant and Stothard, 2008).

**Fig. S5 Dot plot representation of the pairwise alignments of the genome of strain SAT1 against (A) *Nitrosopumilus maritimus* and (B) "*Ca.* Nitrososphaera gargensis".** Alignments were performed on the six-frame amino acid translation of

the genome sequences using the Promer program in the MUMmer 3.0 package. In all plots, a dot indicates a match (of at least six AA) between the two genome sequences being compared, with forward matches colored in red and reverse matches colored in blue.

**Fig. S6 Whole-genome based phylogenomic tree by using composition vector (CV) approach.** This tree was constructed by using composition vector, an alignment-free method, on CVTree3 web server[14,15] (http://tlife.fudan.edu.cn/archaea/cvtree/cvtree3/), with the K-tuple length = 9, the reference genomes were all download from GenBank database.

**Fig. S7 The gene order of ammonia monooxygenase (AMO) of strain SAT1 compared with *Nitrosopumilus .maritimus*, "*Ca.* Nitrosotenuis uzonensis", AOB, "*Ca.* Nitrososphaera gargensis".** Double slash between genes means intervals.

**Fig. S8 Phylogenetic tree showing the relationships of cephalosporin hydroxylase amino acid sequence of strain SAT1 to reference sequences from the GenBank database.** The tree was constructed with the neighbor-joining method using about 250 amino acid positions. Bootstrap values shown at nodes where the value was greater than 50, are based on 1000 trials.

**Fig. S9 Pathway for the synthesis of (A) ectoine and hydroxyectoine, (B) mannosylglycerate, and (C) di-myo-inositol-1, 3'-phosphate.** EctB, L-2,4-diaminobutyrate transaminase; EctA, L-2,4-diaminobutyrate acetyltransferase; EctC, ectoine synthase; EctD, ectoine hydroxylase; MPGS, mannosyl-3-phosphoglycerate synthase; MPGP, mannosyl-3-phosphoglycerate phosphatase; MIPS, myo-inositol-1-phosphate synthase; IPCT, inositol-1-phosphate cytidylyltransferase; DIPPS, di-myo-inositol-1,3-phosphate-1-phosphate synthase; DIPPP, di-myo-inositol-1,3-phosphate-1-phosphate phosphatase. Below enzymes, a red letter "a" inside parenthesis meant that the gene coding for this enzyme was

present in high-salinity group I.1a AOA genomes like *Nitrosopumilus maritimus*, while a letter “b” meant the corresponding gene was present in group I.1b AOA genomes like *“Ca*. Nitrososphaera gargensis*”*, *“Ca*. Nitrososphaera evergladensis*”* and *Nitrososphaera viennensis*. The green cycle on the arrows meant that the gene was identified in the genome of SAT1, while the red cross meant not.

**Fig. S10 The GC-depth plot of primary assembled sequence data.** Region 1 showed the sequences of strain SAT1 and region 2 showed the contaminated bacteria sequences, which were removed from the assembled contigs.

Figure S1

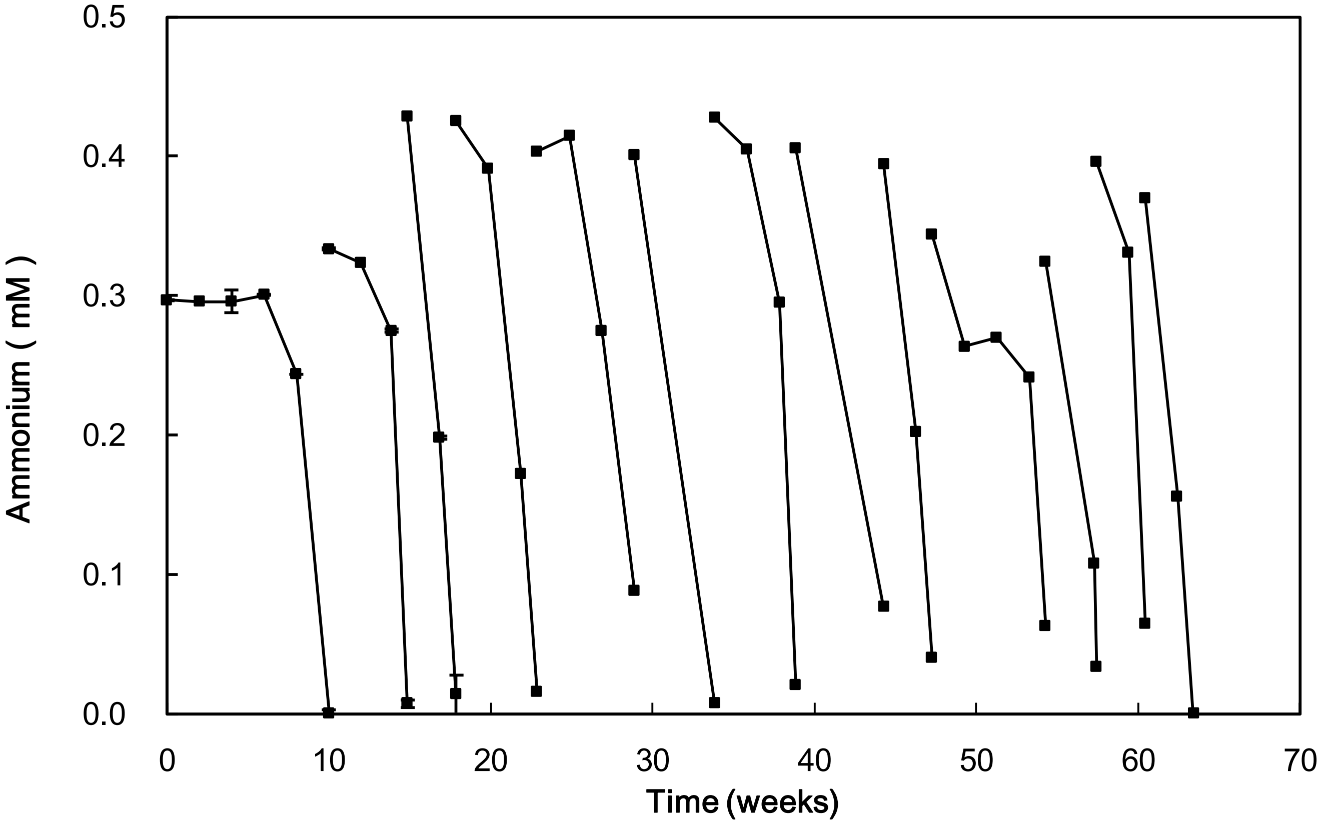

Figure S2

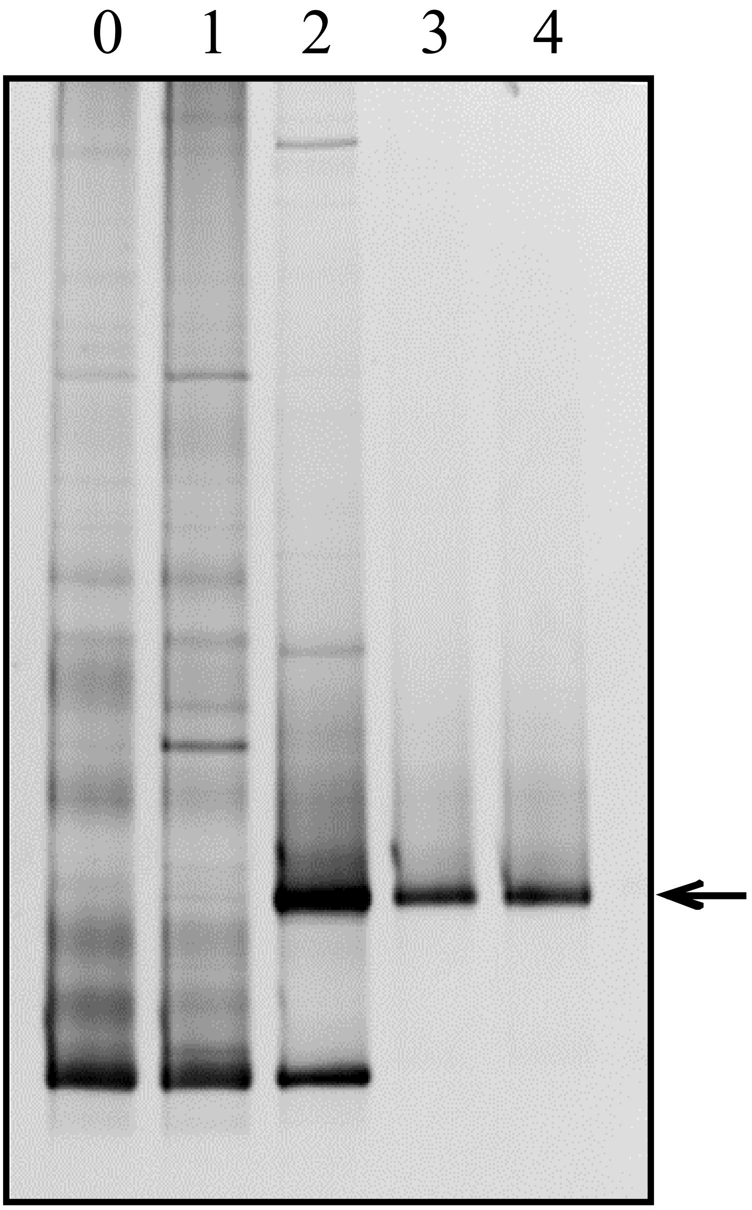

Figure S3

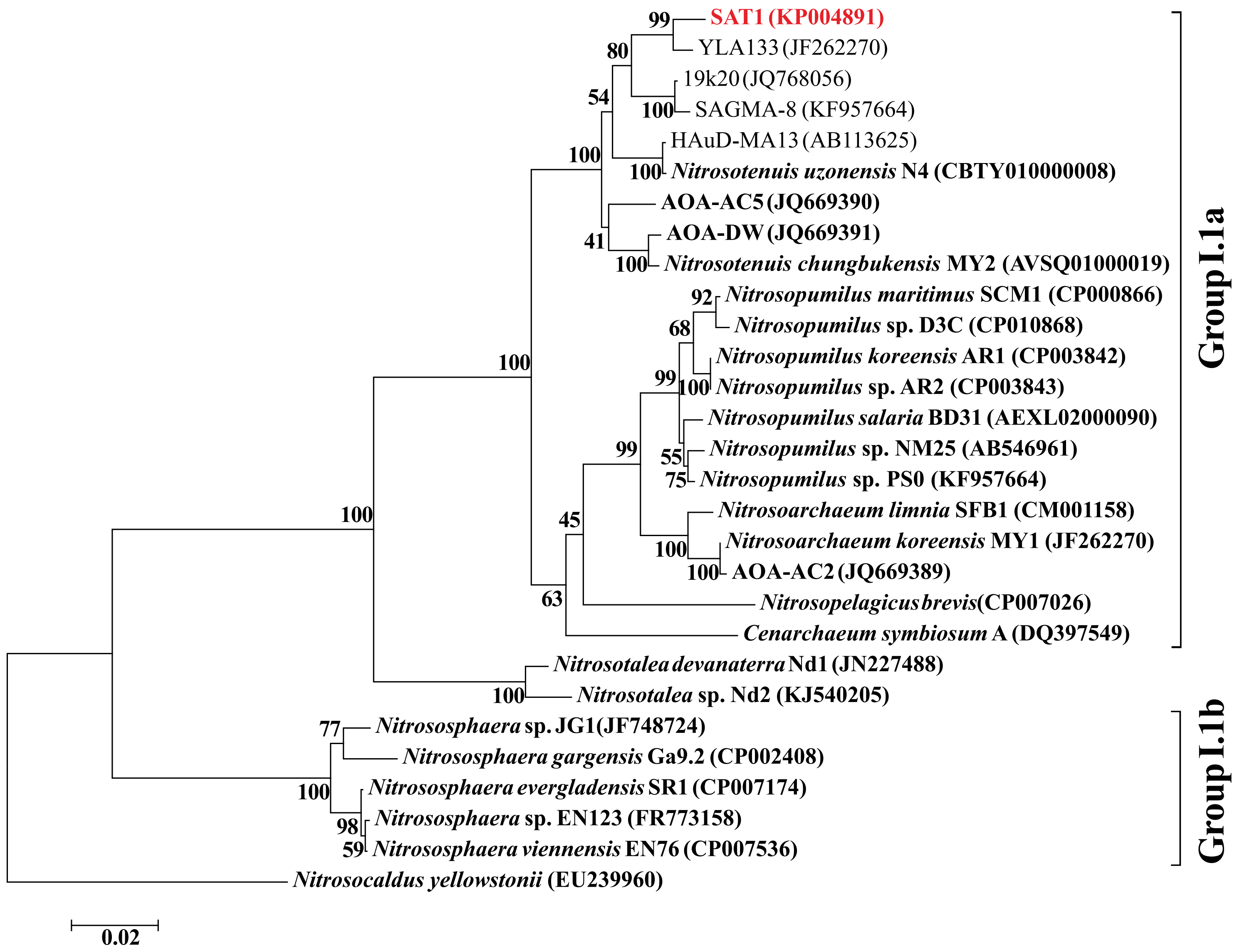

99
SAT1 (KP004891)
80
YLA133 (JF262270)
19k20 (JQ768056)
54
100
SAGMA-8 (KF957664)
HAuD-MA13 (AB113625)
100
100
Nitrosotenuis uzonensis N4 (CBTY010000008)
AOA-AC5 (JQ669390)
AOA-DW (JQ669391)
41
100
Nitrosotenuis chungbukensis MY2 (AVSQ01000019)
92
Nitrosopumilus maritimus SCM1 (CP000866)
68
Nitrosopumilus sp. D3C (CP010868)
100
Nitrosopumilus koreensis AR1 (CP003842)
99
100
Nitrosopumilus sp. AR2 (CP003843)
Nitrosopumilus salaria BD31 (AEXL02000090)
99
55
Nitrosopumilus sp. NM25 (AB546961)
75
Nitrosopumilus sp. PS0 (KF957664)
100
45
Nitrosoarchaeum limnia SFB1 (CM001158)
100
Nitrosoarchaeum koreensis MY1 (JF262270)
100
AOA-AC2 (JQ669389)
63
Nitrosopelagicus brevis (CP007026)
Cenarchaeum symbiosum A (DQ397549)
Nitrosotalea devanaterra Nd1 (JN227488)
100
Nitrosotalea sp. Nd2 (KJ540205)
77
Nitrososphaera sp. JG1 (JF748724)
Nitrososphaera gargensis Ga9.2 (CP002408)
100
Nitrososphaera evergladensis SR1 (CP007174)
98
Nitrososphaera sp. EN123 (FR773158)
59
Nitrososphaera viennensis EN76 (CP007536)
Nitrosocaldus yellowstonii (EU239960)
0.02
Group I.1a
Group I.1b

Figure S4

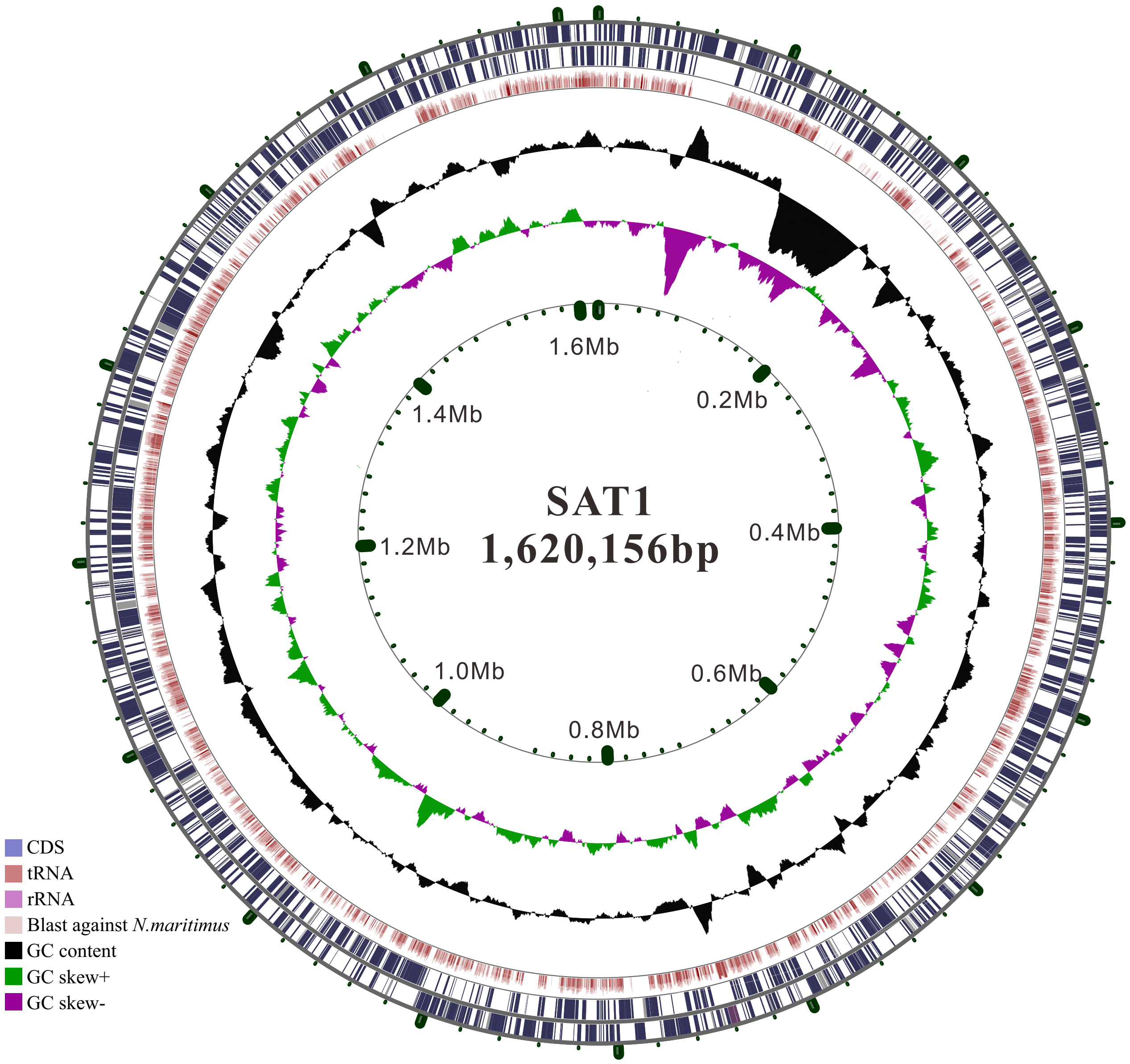
SAT1
1,620,156bp
1.6Mb
0.2Mb
1.4Mb
0.4Mb
1.2Mb
1.0Mb
0.6Mb
0.8Mb
CDS
tRNA
rRNA
Blast against N.maritimus
GC content
GC skew+
GC skew-

Figure S5

(a)

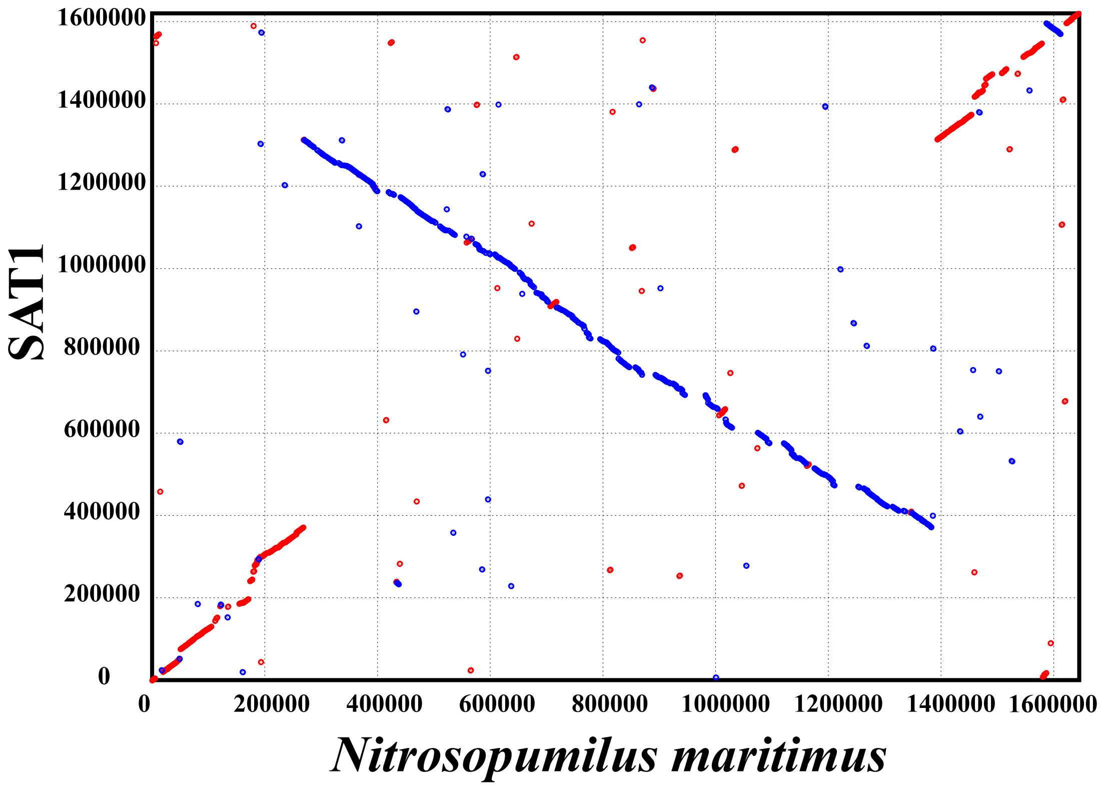
1600000
1400000
1200000
1000000
800000
600000
400000
200000
0
SAT1
0
200000
400000
600000
800000
1000000
1200000
1400000
1600000
*Nitrosopumilus maritimus*

(b)

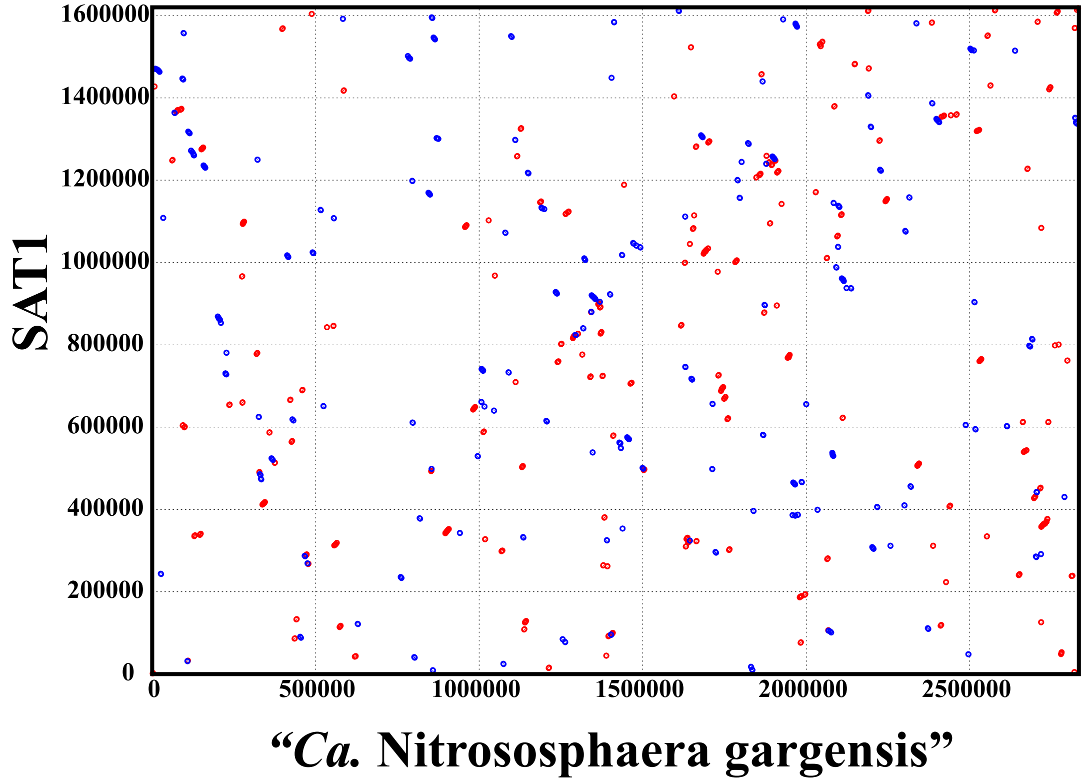
1600000
1400000
1200000
1000000
800000
600000
400000
200000
0
SAT1
0
500000
1000000
1500000
2000000
2500000
"*Ca.* Nitrososphaera gargensis"

Figure S6

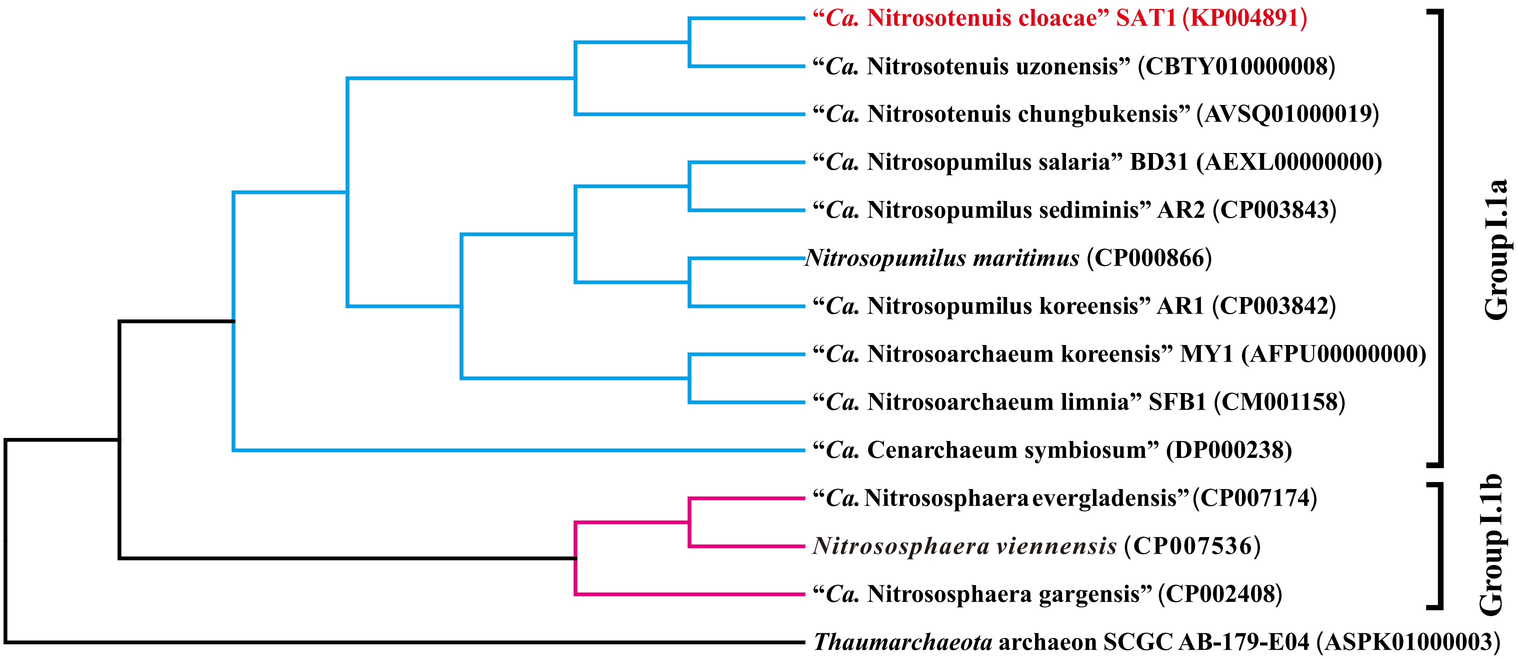

"Ca. Nitrosotenuis cloacae" SAT1 (KP004891)
"Ca. Nitrosotenuis uzonensis" (CBTY010000008)
"Ca. Nitrosotenuis chungbukensis" (AVSQ01000019)
"Ca. Nitrosopumilus salaria" BD31 (AEXL00000000)
"Ca. Nitrosopumilus sediminis" AR2 (CP003843)
Nitrosopumilus maritimus (CP000866)
"Ca. Nitrosopumilus koreensis" AR1 (CP003842)
"Ca. Nitrosoarchaeum koreensis" MY1 (AFPU00000000)
"Ca. Nitrosoarchaeum limnia" SFB1 (CM001158)
"Ca. Cenarchaeum symbiosum" (DP000238)
"Ca. Nitrososphaera evergladensis" (CP007174)
Nitrososphaera viennensis (CP007536)
"Ca. Nitrososphaera gargensis" (CP002408)
Thaumarchaeota archaeon SCGC AB-179-E04 (ASPK01000003)
Group I.1a
Group I.1b

Figure S7

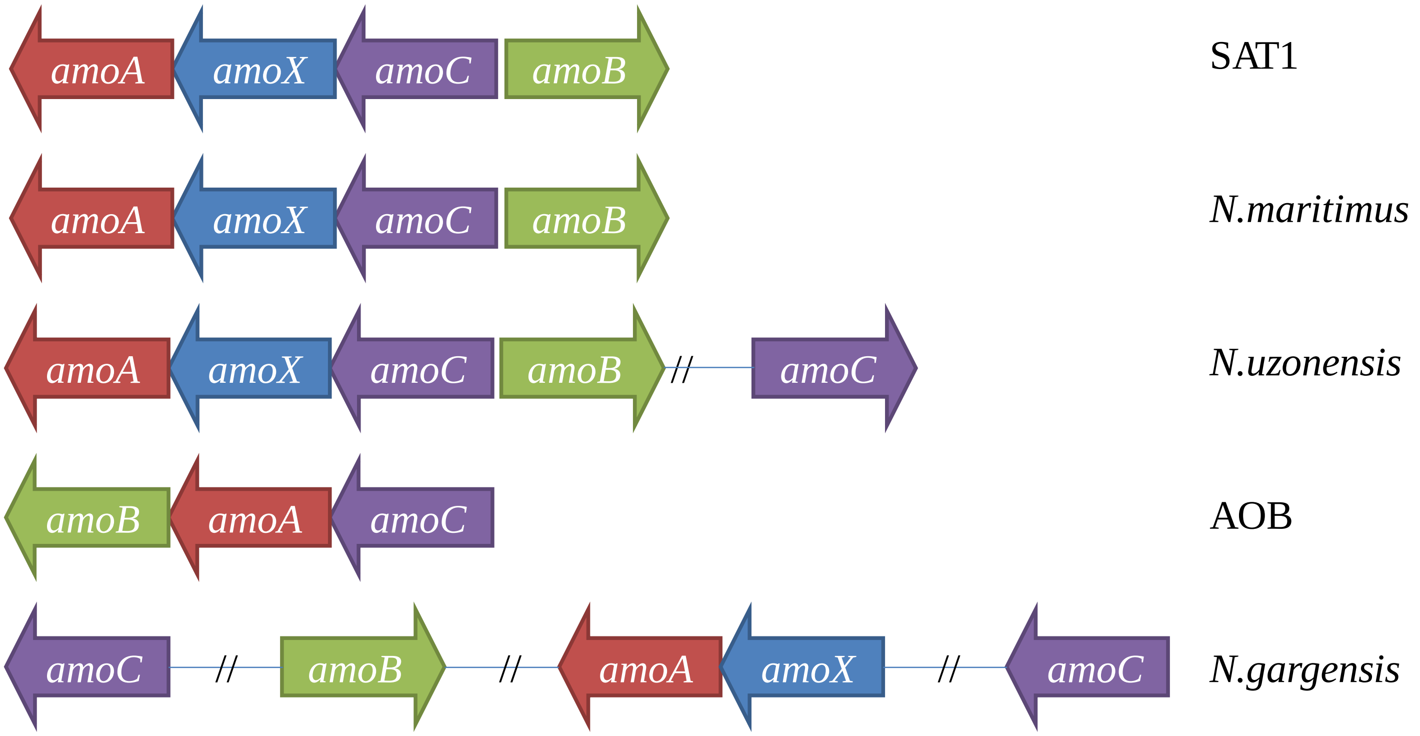
amoA
amoX
amoC
amoB
SAT1
amoA
amoX
amoC
amoB
N.maritimus
amoA
amoX
amoC
amoB
amoC
N.uzonensis
amoB
amoA
amoC
AOB
amoC
amoB
amoA
amoX
amoC
N.gargensis

Figure S8

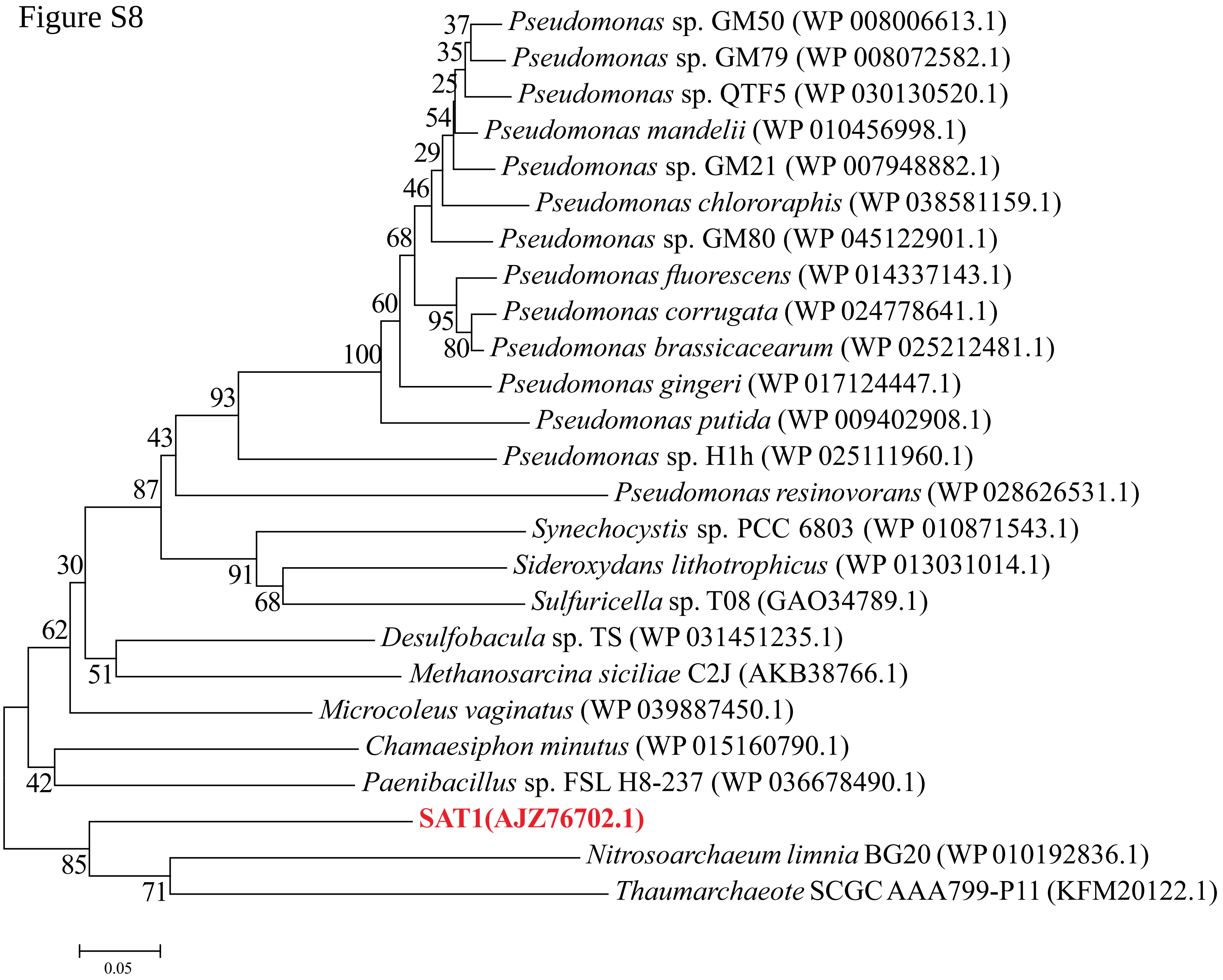

Pseudomonas sp. GM50 (WP 008006613.1)
Pseudomonas sp. GM79 (WP 008072582.1)
Pseudomonas sp. QTF5 (WP 030130520.1)
Pseudomonas mandelii (WP 010456998.1)
Pseudomonas sp. GM21 (WP 007948882.1)
Pseudomonas chlororaphis (WP 038581159.1)
Pseudomonas sp. GM80 (WP 045122901.1)
Pseudomonas fluorescens (WP 014337143.1)
Pseudomonas corrugata (WP 024778641.1)
Pseudomonas brassicacearum (WP 025212481.1)
Pseudomonas gingeri (WP 017124447.1)
Pseudomonas putida (WP 009402908.1)
Pseudomonas sp. H1h (WP 025111960.1)
Pseudomonas resinovorans (WP 028626531.1)
Synechocystis sp. PCC 6803 (WP 010871543.1)
Sideroxydans lithotrophicus (WP 013031014.1)
Sulfuricella sp. T08 (GAO34789.1)
Desulfobacula sp. TS (WP 031451235.1)
Methanosarcina siciliae C2J (AKB38766.1)
Microcoleus vaginatus (WP 039887450.1)
Chamaesiphon minutus (WP 015160790.1)
Paenibacillus sp. FSL H8-237 (WP 036678490.1)
SAT1(AJZ76702.1)
Nitrosoarchaeum limnia BG20 (WP 010192836.1)
Thaumarchaeote SCGC AAA799-P11 (KFM20122.1)
37
35
25
54
29
46
68
60
95
80
100
93
43
87
91
68
30
62
51
42
85
71
0.05

Figure S9

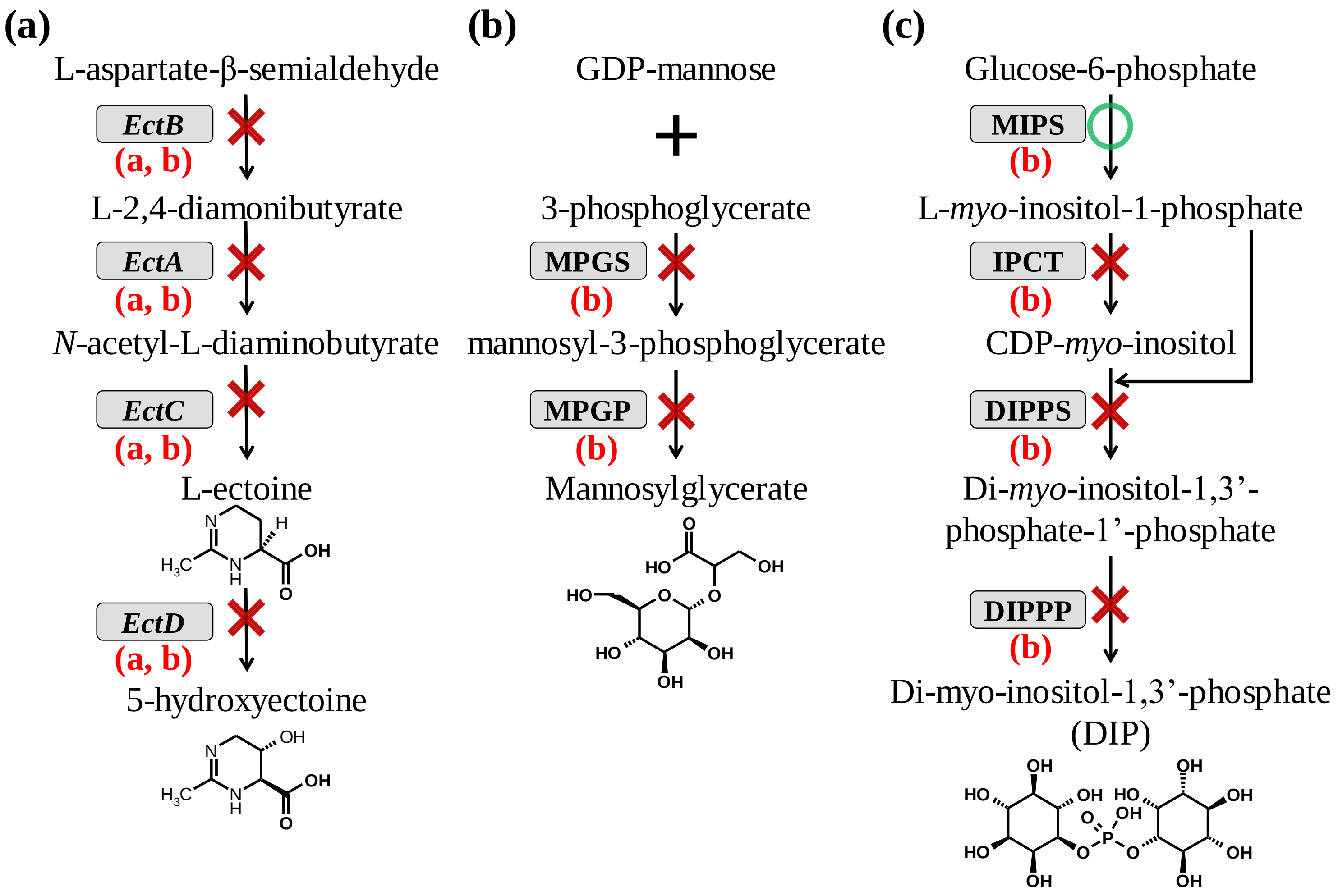

(a)
L-aspartate-β-semialdehyde
EctB
(a, b)
L-2,4-diamonibutyrate
EctA
(a, b)
N-acetyl-L-diaminobutyrate
EctC
(a, b)
L-ectoine
EctD
(a, b)
5-hydroxyectoine
(b)
GDP-mannose
+
3-phosphoglycerate
MPGS
(b)
mannosyl-3-phosphoglycerate
MPGP
(b)
Mannosylglycerate
(c)
Glucose-6-phosphate
MIPS
(b)
L-myo-inositol-1-phosphate
IPCT
(b)
CDP-myo-inositol
DIPPS
(b)
Di-myo-inositol-1,3'-phosphate-1'-phosphate
DIPPP
(b)
Di-myo-inositol-1,3'-phosphate (DIP)

Figure S10

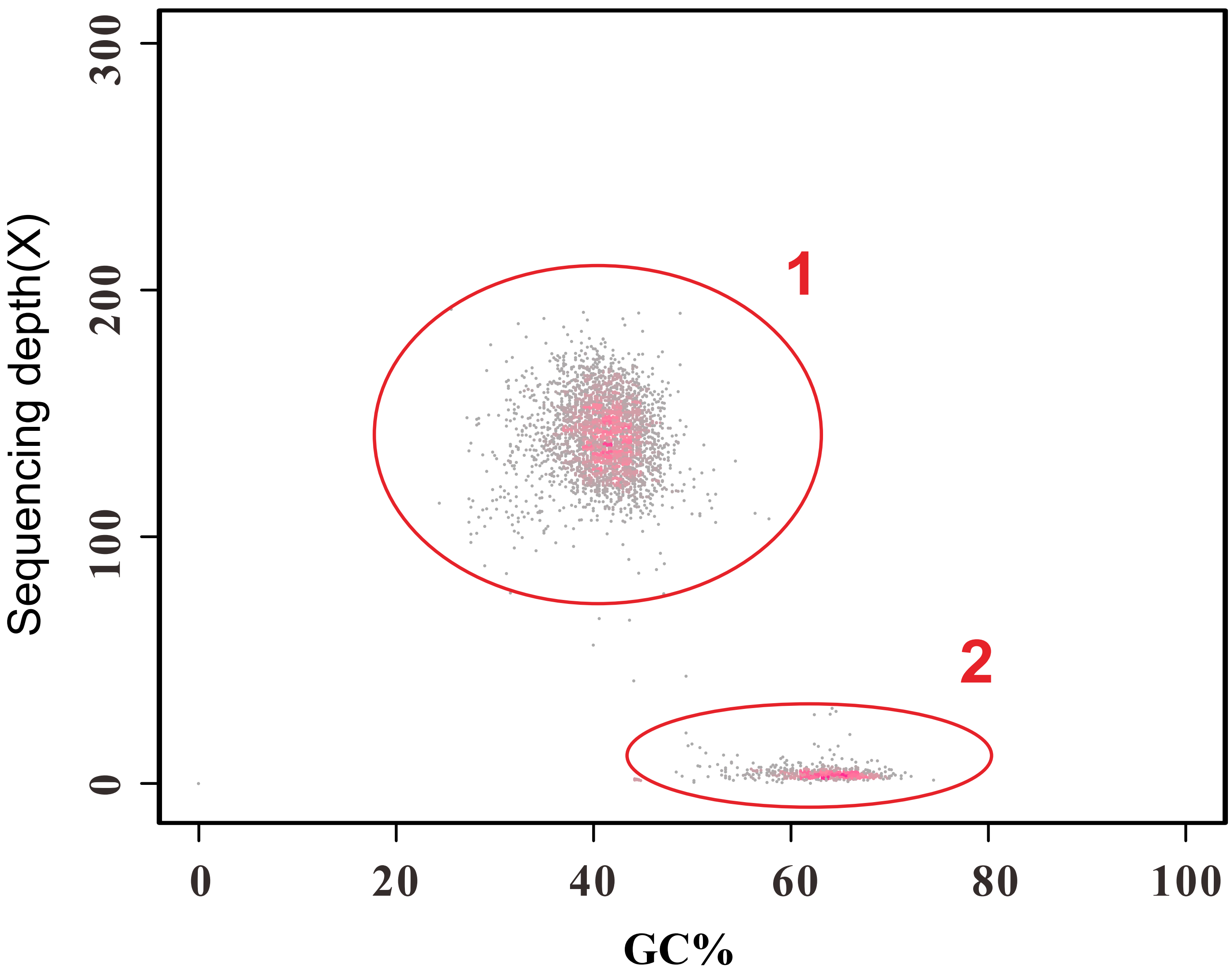

**References:**


1. WEISBURG, W. G., BARNS, S. M., PELLETIER, D. A. & LANE, D. J. 16S ribosomal dna amplification for phylogenetic study. *J Bacteriol*. **173**, 697-703 (1991).
2. DELONG, E. F. Archaea in coastal marine environments. *P Natl Acad Sci USA*. **89**, 5685-5689 (1992).
3. Park, S. J., Park, B. J. & Rhee, S. K. Comparative analysis of archaeal 16S rRNA and amoA genes to estimate the abundance and diversity of ammonia-oxidizing archaea in marine sediments. *Extremophiles*. **12**, 605-615 (2008).
4. Tourna, M. et al. Nitrososphaera viennensis, an ammonia oxidizing archaeon from soil. *P Natl Acad Sci USA*. **108**, 8420-8425 (2011).
5. Tourna, M., Freitag, T. E., Nicol, G. W. & Prosser, J. I. Growth, activity and temperature responses of ammonia-oxidizing archaea and bacteria in soil microcosms E-4902-2011 F-5280-2010. *Environ Microbiol*. **10**, 1357-1364 (2008).
6. MUYZER, G., DEWAAL, E. C. & UITTERLINDEN, A. G. Profiling of complex microbial-populations by denaturing gradient gel-electrophoresis analysis of polymerase chain reaction-amplified genes-coding for 16s ribosomal-rna. *Appl Environ Microb*. **59**, 695-700 (1993).
7. Baker, G. C., Smith, J. J. & Cowan, D. A. Review and re-analysis of domain-specific 16S primers. *J Microbiol Meth*. **55**, 541-555 (2003).
8. Rotthauwe, J. H., Witzel, K. P. & Liesack, W. The ammonia monooxygenase structural gene amoA as a functional marker: Molecular fine-scale analysis of natural ammonia-oxidizing populations. *Appl Environ Microbiol*. **63**, 4704-4712 (1997).
9. Park, H. D., Wells, G. F., Bae, H., Criddle, C. S. & Francis, C. A. Occurrence of ammonia-oxidizing archaea in wastewater treatment plant bioreactors. *Appl Environ Microb*. **72**, 5643-5647 (2006).
10. Wu, Y., Whang, L., Fukushima, T. & Chang, S. Responses of ammonia-oxidizing archaeal and betaproteobacterial populations to wastewater salinity in a full-scale municipal wastewater treatment plant. *J Biosci Bioeng*. **115**, 424-432 (2013).
11. Mussmann, M. et al. Thaumarchaeotes abundant in refinery nitrifying sludges express amoA but are not obligate autotrophic ammonia oxidizers. *P Natl Acad Sci USA*. **108**, 16771-16776 (2011).
12. Bai, Y., Sun, Q., Wen, D. & Tang, X. Abundance of ammonia-oxidizing bacteria and archaea in industrial and domestic wastewater treatment systems. *Fems Microbiol Ecol*. **80**, 323-330 (2012).
13. Gao, J. F., Luo, X., Wu, G. X., Li, T. & Peng, Y. Z. Abundance and diversity based on amoA genes of ammonia-oxidizing archaea and bacteria in ten wastewater treatment systems. *Appl Microbiol Biot*. **98**, 3339-3354 (2014).
14. Qi, J., Wang, B. & Hao, B. I. Whole proteome prokaryote phylogeny without sequence alignment: A K-string composition approach. *J Mol Evol*. **58**, 1-11 (2004).
15. Zuo, G. & Hao, B. CVTree3 web server for whole-genome-based and

alignment-free prokaryotic phylogeny and taxonomy. *Genomics Proteomics Bioinformatics*. **13**, 321-331 (2015).
